# Efficient Radiomics-Based Classification of Multi-Parametric MR Images to Identify Volumetric Habitats and Signatures in Glioblastoma: A Machine Learning Approach

**DOI:** 10.3390/cancers14061475

**Published:** 2022-03-14

**Authors:** Fang-Ying Chiu, Yun Yen

**Affiliations:** 1 Center for Cancer Translational Research, Tzu Chi University, Hualien City 970374, Taiwan; 2 Center for Brain and Neurobiology Research, Tzu Chi University, Hualien City 970374, Taiwan; 3 Teaching and Research Headquarters for Sustainable Development Goals, Tzu Chi University, Hualien City 970374, Taiwan; 4 Ph.D. Program for Cancer Biology and Drug Discovery, College of Medical Science and Technology, Taipei Medical University, Taipei City 110301, Taiwan; 5 Graduate Institute of Cancer Biology and Drug Discovery, College of Medical Science and Technology, Taipei Medical University, Taipei City 110301, Taiwan; 6 TMU Research Center of Cancer Translational Medicine, Taipei Medical University, Taipei City 110301, Taiwan; 7 Cancer Center, Taipei Municipal WanFang Hospital, Taipei City 116081, Taiwan

**Keywords:** annotation, glioblastoma, multi-parametric, machine learning, non-invasive, precision medicine, quantitative imaging, radiomics feature

## Abstract

**Simple Summary:**

Glioblastomas carry a poor prognosis and usually presents with heterogeneous regions in the brain tumor. Multi-parametric MR images can show morphological characteristics. Radiomics features refer to the extraction of a large number of quantitative measurements that describe the geometry, intensity, and texture which were extracted from contrast-enhanced T1-weighted images from anatomical MRI and metabolic features from PET. It also provides a qualitative image interpretation as well as cellular, molecular, and tumor properties. Thus, it derives additional information about the entire tumor volume which is generally of irregular shape and size from routinely evaluated “non-invasive” imaging biomarkers techniques. We demonstrated volumetric habitats and signatures in necrosis, solid tumor, peritumoral tissue, and edema with key biological processes and phenotype features. This provides physicians with key information on how the disease is progressing in the brain and can also give an indication of how well treatment is working.

**Abstract:**

Glioblastoma (GBM) is a fast-growing and aggressive brain tumor of the central nervous system. It encroaches on brain tissue with heterogeneous regions of a necrotic core, solid part, peritumoral tissue, and edema. This study provided qualitative image interpretation in GBM subregions and radiomics features in quantitative usage of image analysis, as well as ratios of these tumor components. The aim of this study was to assess the potential of multi-parametric MR fingerprinting with volumetric tumor phenotype and radiomic features to underlie biological process and prognostic status of patients with cerebral gliomas. Based on efficiently classified and retrieved cerebral multi-parametric MRI, all data were analyzed to derive volume-based data of the entire tumor from local cohorts and The Cancer Imaging Archive (TCIA) cohorts with GBM. Edema was mainly enriched for homeostasis whereas necrosis was associated with texture features. The proportional volume size of the edema was about 1.5 times larger than the size of the solid part tumor. The volume size of the solid part was approximately 0.7 times in the necrosis area. Therefore, the multi-parametric MRI-based radiomics model reveals efficiently classified tumor subregions of GBM and suggests that prognostic radiomic features from routine MRI examination may also be significantly associated with key biological processes as a practical imaging biomarker.

## 1. Introduction

Glioblastoma multiforme (GBM) is the most lethal and aggressive primary brain tumor in adults; with a poor prognosis despite surgical resection combined with radiotherapy and temozolomide administration, the two years survival rate remains at 27% [1]. The 2016 World Health Organization (WHO) began to integrate molecular and genetic profiling to assist in diagnosis [2] and predictive prognosis.

**Figure 1:**
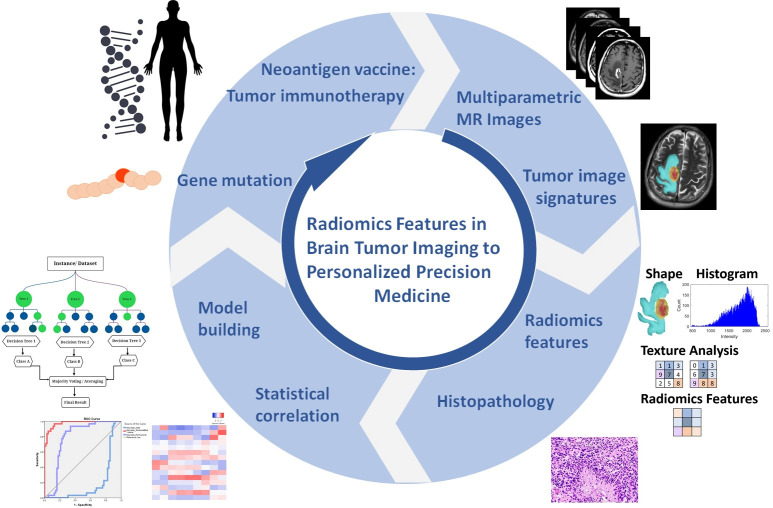
From bench to bedside, translational imaging research can advance to personalized medicine. Clinical implementation of radiomics studies workflow in neuro-oncology includes the following steps: (1) multimodal imaging and biological data labelling; (2) radiomics feature extraction and clinical information integrated model; (3) statistical correlation and machine learning model training; (4) bioinformatics for guiding personalized disease diagnosis, treatment evaluation, and prognostic prediction in precision medicine.

Recently, radiomics has been increasingly exploited to extract and analyze quantitative imaging features. The novel technique becomes more popular in data mining, especially in radiomics features. It can approach tumor phenotypes using thousands of image features that result in the basis for cluster shape, pixel intensity histogram, texture, and diffusion kurtosis analysis covering the entire tumor volume [3]. Several explorative studies have shown the high molecular heterogeneity of gliomas such as the isocitrate dehydrogenase 1 (IDH1) gene, and O-6-methylguanine-DNA-methyltransferase (MGMT) promoter statuses also provide important prognostic information in GBM to advance personalized treatment and translational medicine. From bench to bedside, translational imaging research can advance to personalized medicine. Clinical implementation of radiomics studies workflow in neuro-oncology includes the following steps: (1) multimodal imaging and biological data labelling; (2) radiomics feature extraction and clinical information integrated model; (3) statistical correlation and machine learning model training; (4) bioinformatics for guiding personalized disease diagnosis, treatment evaluation, and prognostic prediction in precision medicine (Figure 1). Several imaging modalities can help diagnosis and metabolite identification such as magnetic resonance imaging (MRI) and positron emission tomography (PET) for anatomical and functional imaging to analyze radiomics features in the classification of the imaging biomarker. GBM tumors illustrate phenotypic features such as necrosis, solid-enhanced tumor, peritumoral tissue, and peritumoral edema. The random forest in machine learning is based on a previous study for model training that analyzed the radiomics features and multiclassification in semantic features [4]. As the underlying drivers of these phenotypes are biological and molecular attributes in nature, this indicated underlying imaging features.

**Figure 2:**
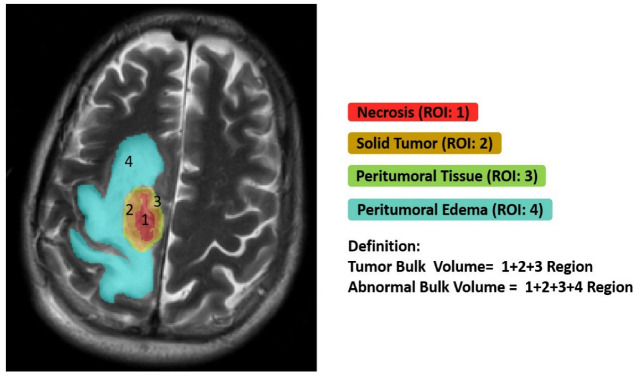
GBM derived from T1-CE MR images for individual measurement in specific necrosis, solid part tumor, peritumoral tissue, and edema regions of right-side frontal lobe volume in a subject. TBV represents the addition of these tumor features. The ABV is represented by hyperintensity extracted from T2-FLAIR images. Edema is the difference of tumor bulk from abnormal bulk volume.

This study implemented semiautomatic annotation from T1-CE MR images for individual measurement in specific necrosis, solid part tumor, peritumoral tissue, and edema regions to extract 1316 features on the raw MR images. We represented radiomic features based on imaging signatures of the heterogeneous GBM tumor tissue parts (Figure 2) and created a radiomic-based model for the semiautomatic annotation of GBM using MRI, ground truth, and machine learning. The features showed the tumor shape elongation of the peritumoral edema region for high-risk groups from T1-CE MR images [4]. Consequently, it derived additional information about the entire tumor volume, which is generally of irregular shape and size, from routinely evaluated “non-invasive” imaging biomarkers techniques and provided qualitative and quantitative information. The novelty of this study is that it showed that classification of multi-parametric image interpretation, radiomics features in quantitative usage of image analysis, and volumetric feature ratios, i.e., tumor bulk volume (TBV) and abnormal bulk volume (ABV) of GBM subregions components are different from other literatures. It will be able to encompass more features concerning intra-tumor heterogeneity in relation to molecular markers and predicting prognosis.

In addition, we presented multi-parametric MRI-based radiomics analysis to identify entirely volumetric habitats as well as ratios of these tumor components, focused on local GBM cohort and the publicly available GBM dataset from The Cancer Imaging Archive (TCIA). To investigate which biological processes derive volumetric tumor phenotype features in this study, we performed a quantitative analysis in two cohorts based on semantic feature in ground truth images using a data analysis. The ratios of volumetric features were significantly associated with various sets. Thus, the feature-based quantitative value in subregions has shown its potential biomarker as an additional source of diagnostic and therapeutic information, and information from routinely acquired MRI exams can also be notably associated with key biological processes that affect the response to chemotherapy in GBM.

## 2. Materials and Methods

### 2.1. Cohort Selection

This study recruited a cohort of 54 patients diagnosed with primary (de novo) confirmed GBM. Two datasets were collected, with 23 patients with GBM from local hospitals as the training data cohort and 31 TCIA datasets as our validation cohort after receiving the institutional review board of Hualien Tzu Chi Hospital approval (IRB110-007-B). 

All local patients suspected of having cerebral GBM based on conventional radiologic findings were enlisted in this prospective study before any treatment (10 women, 13 men; age range, 42–83 years; average age, 62.60 years), and imaging was performed between October 2014 and February 2019. The inclusion criteria were as follows: (1) neuropathological evaluation following surgery or stereotactic biopsy, with all lesions being histopathologically confirmed grade IV glioma; (2) available preoperative MRI consisting of gadolinium-based contrast-enhanced T1-weighted images (T1-CE), T2-weighted images (T2-WI), T2-weighted fluid-attenuated inversion recovery (T2-FLAIR) images, and apparent diffusion coefficient (ADC) images. We retrieved 31 patients with GBM from the publicly available TCIA database as the validation cohort (15 women, 16 men; age range, 18–84 years; average age, 55.13 years). This dataset was released by the BraTS challenge, in which the MRIs were also used to segment GBM patients [5,6].

### 2.2. Imaging Processing & Semantic Annotations

All patients underwent routine MRI scans. The segmentation is performed slice-wise, where the input data include the T1-CE, T2-WI, T2-FLAIR, and ADC MR images of each patient. All methods were applied to the T1 post-contrast images using default parameters, except for machine learning models that have no tunable parameters. This study implemented semiautomatic annotation for extraction radiomics features as described. Consequently, we made ground truth segmentation masks from tumor subregions delineation and semantic features which were manually generated using the multi-parametric MR images following a specific given annotation protocol. Four subregions were delineated on the tumor imaging, namely, the complete tumor extent also referred to as: (1) the core area (necrotic tumor region), (2) solid-enhanced tumor, (3) peritumoral tissue, and (4) peritumoral edema. Additionally, TBV and ABV represented these tumor features (illustrated in Figure 2). The protocol used for annotating the tumor structures was represented in detail in those two BraTS literatures.

### 2.3. Extraction of Radiomics Features

Radiomics features were extracted as described previously [4]. A total of 1316 features on the raw MR images were selected for each annotated area, including 32 geometry features (size- and shape-based features), 128 intensity histograms (64 first-order and 64 histogram features of local binary pattern (LBP)), 132 texture features (44 gray-level run-length matrix (GLRLM) features and 88 gray-level co-occurrence matrix (GLCM) features), and 1024 scale-invariant feature transform features. 

The manually corrected segmentation features were extracted from four subregions: necrosis, solid part, peritumoral tissue, and peritumoral edema. The feature dataset was divided into three groups: (1) geometry: the three-dimensional morphological characteristics of the tumor, (2) histogram: the first-order statistics computed from pixels and voxel intensities, and (3) texture: second- and high-order spatial distributions of the intensities in the image. The texture features were extracted using several methods, including features based on the GLCM and GLRLM. We adopted the LBP histogram for texture; this summarizes the texture characteristics and patterns of a surface into a histogram, which can be used as input for pattern classification. All the features were combined and used as the input radiomic features for the machine learning model. The workflow of the proposed method presented in this study for non-invasive practical imaging biomarkers in precision medicine is demonstrated in Figure 1.

### 2.4. Training and Validation in Machine Learning Algorithms

According to previous interpretation [4], using random forest achieves the best accuracy 95.8%, precision 97.3%, recall 97.3%, and F1-score 0.898 in peritumoral tissue. The area under the curve (AUC) is the best classification (greater than 95%) in all subregions on which to stratify the training data to improve machine-learning-based brain tumor region classification. For random forest, we used the number of features ranging from 100 to 2000 (step size = 100), and maximum depth values ranging from zero to 110 (step size = 10). In consequence of the limited data, leave-one-out cross-validation was used to validate the overall performance. The accuracy of classification was evaluated with majority vote (i.e., a threshold cutoff of 50%) [7].

### 2.5. Statistical Analysis

All the statistical data analysis in this study was performed with SPSS software with implemented statistics analysis (IBM Corp, Armonk, NY, USA). The differences in patient cohort, tumor size, volume, and training and validation datasets were evaluated using an ANOVA test for differences in average volume, mean distribution, and 95% confidence interval mean. A *p* value < 0.05 was considered to be statistically significant. All differences were significant by repeated-measures analysis of variance with Bonferroni correction.

## 3. Results

### 3.1. Ground Truth Segmentation and Identification of Tumor Habitats on MRI

To investigate which biological processes and morphological characteristics on multi-parametric MR images drive volumetric tumor phenotype features in GBM, we performed the qualitative and quantitative interpretation based on semantic features in GBM subregions. It displays tumor habitats which are color-coded and overlaid on T2-FLAIR annotated images for the ground truth from GBM. Different GBM regions were accurately labeled into four regions of interest and joint intensity color maps on T2-FLAIR: necrosis (red), solid part tumor (brown), peritumoral tissue (green), and peritumoral edema (turquoise) as shown in Figure 2 and correspondingly in Table 1.

### 3.2. Classification of Multi-Parametric MR Images to Identify Volumetric Habitats and Signatures

**Figure 3:**
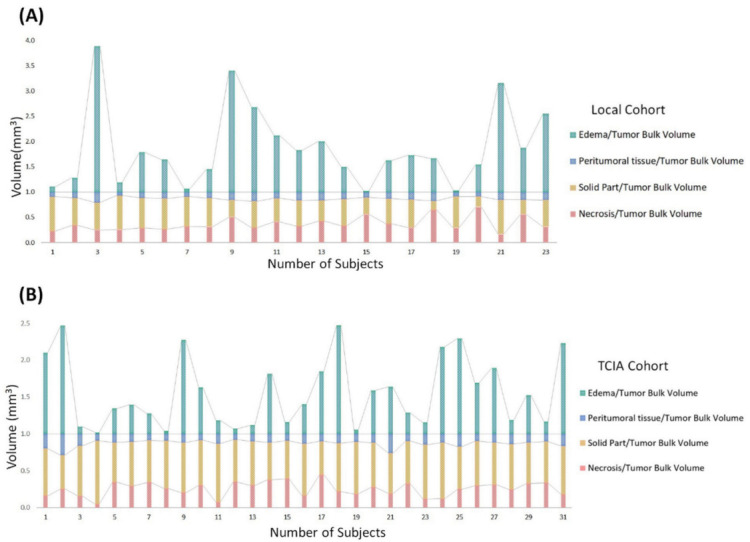
Stacked column chart reveals the size distribution of volumetric tumor features across subregions of GBM in (**A**) local and (**B**) TCIA cohort. Compared to the tumor bulk volume, edema had the largest median size across all subregions. Solid part tumor and peritumoral tissue showed more consistent areas than other regions. Size variation of volumetric feature areas other than edema was generally low across subregions.

**Figure 4:**
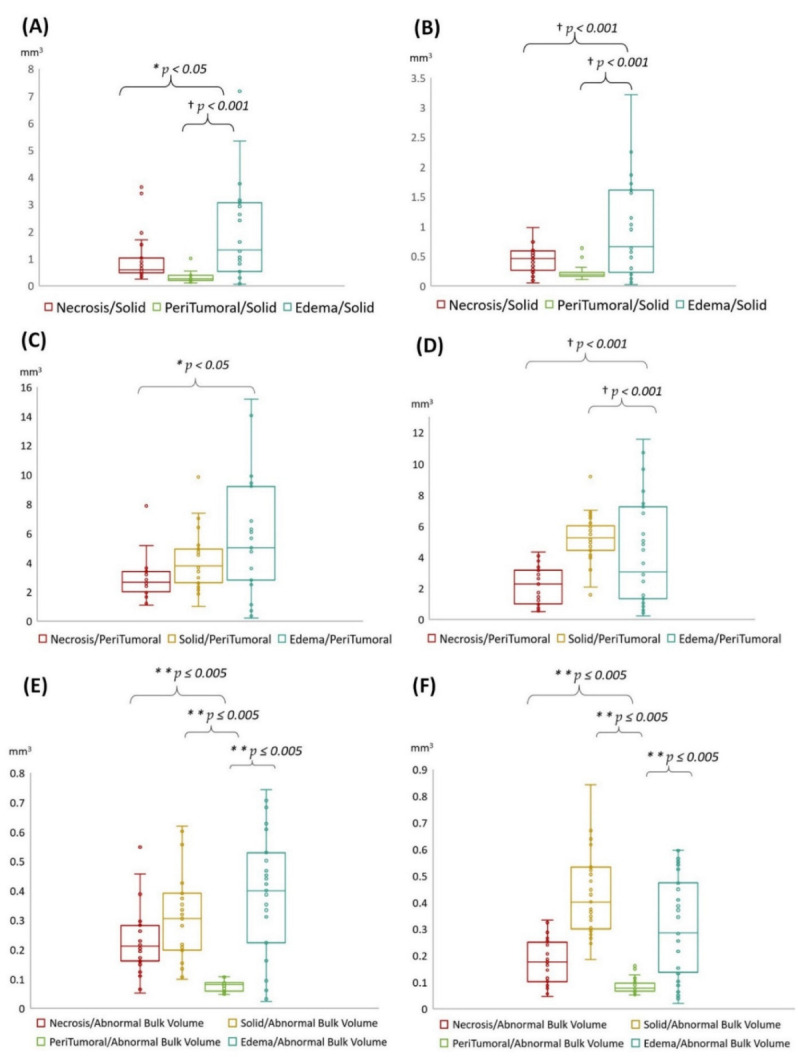
Box-and-whisker plot shows the volumetric size distribution of subregion ratio types for two cohorts in local (**A,C,E**), and TCIA (**B,D,F**) on multi-parametric MR images. (**A**–**F**) show representative different subregions to solid tumor, peritumoral tissue, and abnormal bulk volume ratio, respectively. Repeated-measures ANOVA with Bonferroni correction. Statistically significant difference between each group *p* < 0.05 (*), *p* ≤ 0.005 (**), and *p* < 0.001 (†).

We quantified the following volumetric features in GBM based on multi-parametric MR images: necrosis, solid part tumor, peritumoral tissue, and peritumoral edema. We also quantified TBV and ABV for comparison in tumor subregions. In addition, we calculated the following ratios mainly to explore combined T1-CE and T2-FLAIR signals: (1) four subregions (necrosis, solid part tumor, peritumoral tissue, and peritumoral edema) to TBV ratio (Figure 3). (2) Necrosis, peritumoral tissue, and peritumoral edema to solid tumor ratio (Figure 4A,B). (3) Necrosis, solid part tumor, and peritumoral edema to peritumoral tissue ratio (Figure 4C,D). (4) Four subregions to ABV ratio (Figure 4E,F). The areas of the tumor subregions that these features correspond to are highlighted in Figure 2 as well.

### 3.3. Biological Processes Underlying Volumetric Features 

**Figure 5:**
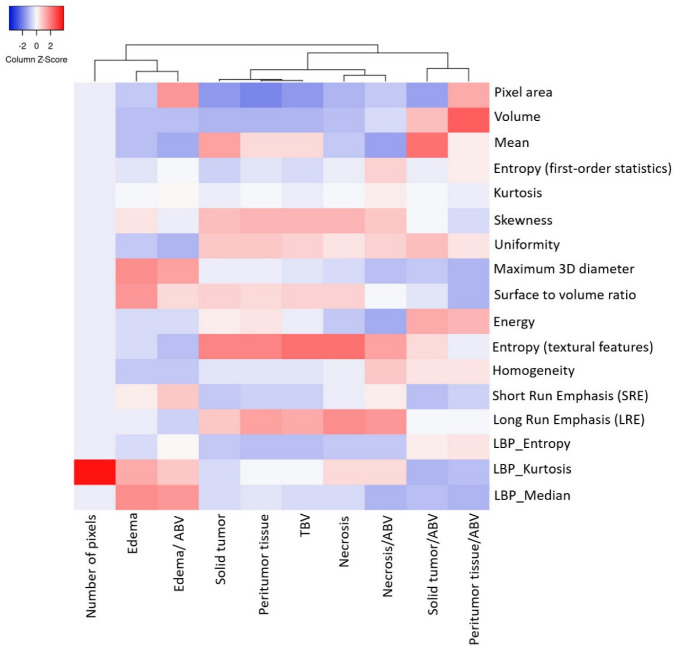
The heatmap manifests the absolute quantification radiomics feature values with high to low feature values in red to blue. The data showing hierarchical cluster analyzed using MR radiomics features and cluster distance implied the order in which clusters were associated.

Volumetric features were significantly associated with various sets of tumor phenotype features and biological processes in local (Figure 4A,C,E) and TCIA (Figure 4B,D,F) cohort studies. Edema was mainly enriched for homeostasis. The proportional volume size of the edema was about 1.5 times larger than the size of the solid part tumor. The volume size of the solid part was approximately 0.7 times in the necrosis area (Table 2 and Figure 4A,B). The peritumoral tissue is a small region in GBM; for comparison, see other subregions (Table 3 and Figure 4C,D). However, these features were enriched for biological processes in immune response and apoptosis [8]. Figure 4C,D show the volumetric prototype plot which is underlain by the original tumor habitat that contains the tumor subregions. The outlier area shows the data point that differs significantly from other observations. The ABV was associated with a larger number of biological processes than the original features (Table 1 and Figure 4E,F). The quantitative image analysis collected GBM identification, volumetric values, and texture features to validate the capability of multi-parametric MR imaging (Figure 5). The heatmap shows that solid tumor, peritumor tissue, and necrosis could be clustered together with features of skewness, uniformity, and textural features possessing linkage. 

### 3.4. Statistical Results

Four subregions (i.e., necrosis, solid part tumor, peritumoral tissue, and peritumoral edema) and ratio compared (i.e., TBV and ABV) were all significantly different in value of volumetric features. Analysis of variance was used to account for repeated measurements within different tumor subregions. A *p* value < 0.05 was considered to be statistically significant after correction for multiple comparisons using Bonferroni correction (Table 1, Table 2 and Table 3 and Figure 4). Notably, statistical ABV ratio comparisons were strong, significantly (** *p* ≤ 0.005) in agreement with local patients and TCIA database as our validation cohort to carry out a pilot study in Figure 4E,F.

## 4. Discussion

Medical imaging can be considered a practical novelty in technology and science. It is used in clinical practice to aid decision making for diagnosis and treatment in disease. This novel technique has become more popular in data mining, especially in radiomics feature which is an emerging field that transforms imaging raw data into a high-dimensional mineable feature space using a large number of automatically extracted data-based features algorithms [4,9,10,11,12,13,14,15,16,17]. It can approach tumor phenotypes using thousands of image features that result in the basis for pixel intensity histogram, cluster prominence, cluster shade, inertia, geometry, texture, and diffusion kurtosis analysis covering the entire tumor volume [3]. This usage provides the extraction of quantitative features from medical images such as CT, MRI, or PET. Hence, it provides supplementary, potentially applicable diagnostic and therapeutic guidelines for clinical decision making. Moreover, the radiomics features can be used alone or combined with other clinical or histomolecular parameters to generate predictive or prognostic mathematical or machine learning models which can then be applied for various significant diagnostic indications in neuro-oncology [18]. Non-invasive biomarkers can reflect the underlying tumor’s habitats and signatures to differentiate between treatment-related changes and local brain tumor recurrence, and can offer quantitative information to guide therapy for personalized disease diagnosis, treatment evaluation, and prognostic prediction in precision medicine. 

Several literatures have proposed to evaluate the feature-based different modality in patients with GBM. The feature findings were compared with MRI and PET/MRI based on radiomics analysis (Table 4) [4,9,10,11,12,13,14,18,19,20,21]. The multi-parametric MR images analysis and the use of artificial-intelligence–based approaches for image-based analysis and construction of expectation algorithms manifest a new epoch for noninvasive biomarker discovery. Additionally, PET/MRI provides the structural information obtained from conventional MRI and tumor metabolism from PET. A variety of radiotracers are obtainable for patients with brain tumors. Particularly, there is diagnostic value in amino acid PET tracers; (O-(2-[^18^F]fluoroethyl)-L-tyrosine, ^18^F-FET) is a promising tracer that has manifested convincing results, especially in the diagnostics of brain tumors [22,23,24,25,26]. The combination of radiomics and metabolic feature will raise significant signatures in prediction of molecular markers, differentiation of treatment-related changes, and radiation necrosis from tumor recurrence in brain metastases that are necessary for essential diagnostic information in neuro-oncology (Table 4).

Semiautomatic segmentation represents the classification for response assessment using volumetric measurements that may capture tumor shape and geometry more accurately; this is particularly useful for GBMs, which are often irregularly shaped. In addition, large-scale studies have proven the benefits of using volumetric-based radiomics features for tumor segmentation compared with intricate verification approaches [4,8]. To ensure the reliability of quantitative imaging features, tumor contouring by manual delineation is used to separate four different regions of tissues, individually. This article shows different ways to compare MRI and PET/MRI based on radiomics analysis, feature types, and performance in different classified methods in Table 4, accordingly.

First, different GBM regions were accurately labeled into four ROIs and joint intensity color-maps on T2-FLAIR in heterogeneous tumor regions (Figure 2); on the contrary, several literatures only showed segmentation in three regions (i.e., enhancing tumor, non-enhancing tumor, edema) for reproducible clustering points to three distinct imaging subtypes (Table 4).

Second, our investigation provided qualitative image interpretation in GBM subregions and radiomics features in quantitative usage of image analysis, as well as volumetric feature ratios (i.e., ABV and TBV) of these tumor components. The proportional volume size of the edema was about 1.5 times larger than the size of the solid part tumor, which means the edema was mainly enriched for homeostasis. Whereas the solid part was approximately 0.7 times in the necrosis area. Consequently, the multi-parametric MRI-based radiomics model revealed quantitative and qualitative information in four subregions of GBM. The novelty of this study was that we could mine images to discover patterns that predict biological characteristics of the radiomics features from these points of views.

Third, computational features were extracted using annotated imaging, particularly those that quantify shape and margin sharpness. Both color-coded semantics and features were derived from multimodal MR radiomics to analyze GBM texture features and compare tumor characteristics such as entropy and homogeneity and correlate them with spatial-habitat imaging. All features were combined and fed into the machine learning algorithms for accessing and interoperating with the semantic content in medical imaging. The AUC is the best classification in all subregions on which to stratify the training data to improve machine-learning-based brain tumor region classification in comparison to other studies.

To investigate which biological processes derive volumetric tumor phenotype features in this study, we performed a quantitative analysis in two cohorts based on semantic features in ground truth images using a data analysis. We compared these results to radiomics features; in our analysis, the ratios of volumetric features were significantly associated with various sets. The results of this study outline issues of major concern:

(1) Edema was mainly enriched for homeostasis. The proportional volume size of the edema was about 1.5 times larger than the size of the solid part tumor. The volume size of the solid part was approximately 0.7 times in the necrosis area. Therefore, the multi-parametric MRI-based radiomics model efficiently classified tumor subregions of GBM, and there are implications that prognostic radiomic features from routine MRI examination may also be significantly associated with key biological processes that affect response to chemotherapy in GBM as a practical imaging biomarker.
(2) The volumetric prototype plot is underlain by original tumor habitat that contains the tumor subregions. Generally, many more biological processes were significantly associated with the feature ratios, usually showing a trend towards a mix of pathways associated with the individual features [8]. In our investigation, volumetric feature ratios (i.e., ABV and TBV) were associated with a larger number of biological processes than the original features. Hierarchical cluster analysis using a subset of features has identified five distinct clusters according to the different volumetric habitats and features. The different types of cluster in entropy (textural feature), long run emphasis, skewness, uniformity, and LBP are correlated with classification among tumor subregions in solid part, peritumoral, necrosis, and edema, respectively.
(3) Contrast enhancement was enriched for signal transduction and protein-folding processes. The peritumoral tissue is a small region in GBM in comparison with other subregions. However, these features were enriched for biological processes in immune response and apoptosis from spatial immunoprofiling while the abundance and phenotype of tumor infiltration lymphocytes are closely linked with clinical survival [8,27]. That reflected the regulation of gene expression such as autophagy gene in necrosis [28], vascular endothelial growth factor gene in peritumoral tissue [29], and angiogenesis gene in edema [30,31] with pathological and molecular features of GBM [32]. Moreover, aquaporin 4 (AQP4) contributes to extended tumor cell migration, possibly passing through increasing water permeability and implication of AQP4 in tumor edema [33,34].
(4) Biological features correlate with corresponding MR imaging sequences because different MR imaging sequences come with diverse clinical imaging protocols. Quantitative features provide tumor microenvironment, spatial characteristics, distinguishing of molecular subtypes, survival predictor corresponding to LBP and histogram of oriented gradients, scale-invariant feature transform, histogram of contrast-enhanced tumor MRI, and contrast information between co-occurring subregions, respectively. Consequently, selection of MR imaging sequences can directly affect image feature definition and the corresponding biological interpretation [35]. Particularly, more biologically significant MRI sequences such as diffusion- and perfusion-weighted MRI have been shown to outperform radiomics models based on conventional MRI [4,9]. These approaches should be taken into account in future research as they will be able to encompass more features concerning intratumor heterogeneity and have shown improved performance in relation to molecular markers and predicting prognosis [9,36].
(5) We illustrated radiomic features based on imaging signatures of the heterogeneous GBM tumor tissue parts (Figure 2) and created a radiomic-based model for the semiautomatic annotation of GBM using MRI, ground truth, and machine learning [4]. The performance of radiomics has been demonstrated when features are extracted from distinct tumor areas such as active tumor, necrosis, and edema, separately. It will be much better for specific tumor areas in clinical applicability [36,37,38].

Several aspects have crucial challenges for accurate features. This plays a pivotal role when it comes to extraction of radiomics-based imaging signature and the large number of quantitative measurements in two cohorts. 

The first challenge is variability in tumoral extraction: tumor heterogeneity causes and consequences. In 2010, Marusyk et al. [39] proposed that the greater number of human tumors illustrate heterogeneity in many morphological and physiological features, such as expression of cell surface receptors and proliferative and angiogenic potential. To a substantial extent, this heterogeneity might be attributed to morphological and epigenetic plasticity, but there is also strong evidence for the coexistence of genetically divergent tumor cell clones within tumors. We have to address this point; it would be advantageous when using semiautomatic segmentation to decide GBM shape and geometry more accurately. Furthermore, GBMs are characterized by heterogeneous angiogenesis, cellular proliferation, cellular invasion, and apoptosis, which translate into diversifying grades of necrosis, solid enhancing tumor, peritumoral tissue, and peritumoral edema. In particular, irregular rim enhancement surrounding the necrosis is the most complicated form for imaging annotation. It was found to be prominently associated with unmethylated MGMT promoter status, which is known to be a biomarker for response to temozolomide and survival. By contrast, semiautomatic segmentation would be advantageous to determine GBM shape in each patient. In this perspective, this needs to be compared with different neuroimaging biomarkers for quantitative and qualitative image interpretation of tumoral heterogeneity surrogates as a reference [40,41]. 

The second challenge is generalizability: The multi-parametric radiomics-based approach provides extraordinary potential and possibility to contribute to an improved diagnosis and treatment guideline in neuro-oncology. However, a few barriers must be overcome before this method can be successfully integrated into clinical routine. The reproducibility and transferability of the developed models and the underlying radiomics features should also be improved [18]. These parameters often depend on many different factors, such as the image quality and the pre- and post-processing steps. Similarly, the independent evaluation of the developed model to validate the dataset test is important. Moreover, it needs to be compared with different neuroimaging biomarkers for quantitative and qualitative image interpretation of tumoral heterogeneity surrogates as a reference. Furthermore, for a successful translation of radiomics into clinical routine, model validation in large-scale cross-institutional datasets is of prominent significance. This hindrance could be conquered by multi-center cooperation and large-scale datasets from phase II or III clinical trials [18,42].

The third challenge is the small sample size of limitation. Our study focused on semantic and volumetric phenotype features such as the radiomics-based definitions of imaging phenotypes that are available. However, we attempted to compare our results with real-world clinical experience and across different types of study for correlation with radiomics feature accuracy and efficiency related to specific regions of GBM. We expect that in future studies the prognostic performances will increase cohorts with even larger numbers of samples and generalizability image processing will become available for GBM.

The development of bioinformatics technology has expedited the identification of neoantigens as shown in Figure 1. Neoantigen vaccine is an emerging tumor immunotherapy which is derived from tumor-specific protein-coding mutations. It can generate tumor-specific antigens and highly expressed immunogenic agents as they are not expressed in normal tissues which are exempt from central tolerance. They can activate cell surface proteins CD4+ (helper) and CD8+ (killer) T cells to generate immune response and have the potential to become new targets of tumor immunotherapy [43,44,45]. Zhang et al. [46] show that the neoantigen quality fitness model stratifies GBM patients with more profitable clinical outcome and, together with CD8+ T lymphocytes tumor infiltration, identifies a GBM subgroup with the longest survival, which shows discriminate transcriptomic and genomic features. From this point of view, neoantigen vaccine will be helpful in target therapy for treatment evaluation in precision medicine.

## 5. Conclusions

The present study leveraged radiomic features based on imaging habitats and signatures of the heterogeneous GBM tumor tissue parts and created a volumetric radiomic-based model for the semiautomatic annotation of GBM using MRI, ground truth, and machine learning. Volumetric features were significantly associated with various sets of tumor phenotype features and biological processes. In this perspective, non-invasive biomarkers can reflect underlying tumors’ habitats and signatures to provide quantitative information to lead therapy for guiding personalized disease diagnosis, treatment evaluation, and prognostic prediction in precision medicine.

## Figures and Tables

**Table 1:** Statistical abnormal bulk volume (ABV) comparisons between local patients with GBM and TCIA database as our validation cohort to carry out a pilot study.

	1. Local Patients with GBM		2. TCIA Database with GBM	
Parameter	Mean ± SD (Min.–Max.)	95% Confidence Interval-Mean (Lower–Upper Bound)	Mean ± SD (Min.–Max.)	95% Confidence Interval-Mean (Lower–Upper Bound)
Necrosis/ABV	0.23 ± 0.12 (0.05–0.55)	0.18–0.28 **	0.17 ± 0.83 (0.05–0.33)	0.14–0.20 **
Solid/ABV	0.31 ± 0.16 (0.10–0.62)	0.24–0.38 **	0.43 ± 0.15 (0.18–0.84)	0.37–0.49 **
Peritumoral tissue/ABV	0.08 ± 0.02 (0.05–0.11)	0.07–0.08	0.09 ± 0.03 (0.05–0.16)	0.08–0.10
Edema/ABV	0.39 ± 0.21 (0.02–0.74)	0.30–0.48 **	0.31 ± 0.19 (0.02–0.60)	0.24–0.38 **

All differences were significant by repeated-measures analysis of variance with Bonferroni correction. Differences compared with peritumoral tissue/ABV ratio at *p* ≤ 0.005 (**). SD, standard deviation; Min, minimum; Max, maximum.

**Table 2:** Statistical solid tumor comparisons between local patients with GBM and TCIA database as our validation cohort to carry out a pilot study.

	1. Local Patients with GBM		2. TCIA Database with GBM	
Parameter	Mean ± SD (Min.–Max.)	95% Confidence Interval-Mean (Lower–Upper Bound)	Mean ± SD (Min.–Max.)	95% Confidence Interval-Mean (Lower–Upper Bound)
Necrosis/Solid	0.97 ± 0.91 (0.24–3.64)	0.57–1.36 *	0.44 ± 0.21 (0.05–0.98)	0.36–0.51 †
Peritumoral tissue/Solid	0.31 ± 0.19 (0.10–1.00)	0.23–0.39 †	0.22 ± 0.10 (0.11–0.64)	0.18–0.26 †
Edema/Solid	1.98 ± 1.80 (0.05–7.17)	1.20–2.76	0.96 ± 0.82 (0.02–3.22)	0.66–1.27

All differences were significant by repeated-measures analysis of variance with Bonferroni correction. Differences compared with edema/solid ratio at *p* < 0.05 (*), *p* < 0.001 (†). SD, standard deviation; Min, minimum; Max, maximum.

**Table 3:** Statistical peritumoral tissue comparisons between local patients with GBM and TCIA database as our validation cohort to carry out a pilot study.

	1. Local Patients with GBM		2. TCIA Database with GBM	
Parameter	Mean ± SD (Min.–Max.)	95% Confidence Interval-Mean (Lower–Upper Bound)	Mean ± SD (Min.–Max.)	95% Confidence Interval-Mean (Lower–Upper Bound)
Necrosis/Peritumoral tissue	2.94 ± 1.42 (1.09–7.88)	2.33–3.56	2.20 ± 1.18 (0.50–4.32)	1.76–2.63
Solid/Peritumoral tissue	4.12 ± 2.03 (1.00–9.85)	3.25–5.00	5.16 ± 1.49 (1.57–9.17)	4.61–5.70 †
Edema/Peritumoral tissue	5.89 ± 4.34 (0.21–15.15)	4.01–7.76 *	4.25 ± 3.31 (0.21–11.55)	3.04–5.46 †

All differences were significant by repeated-measures analysis of variance with Bonferroni correction. Differences compared with necrosis/ peritumoral tissue ratio at *p* < 0.05 (*), *p* < 0.001 (†). Note: SD, standard deviation; Min, minimum; Max, maximum.

**Table 4:** The comparison of feature findings with MRI and PET/MRI based on radiomics analysis.

Study	Theme	MRI Sequences PET Tracer	Feature Type	Classification Method	Performance (Training)
MRI					
Chiu et al. (2021) [4]	Efficiently classify tumor subregions of GBM for prognostication with key biological processes	T1-CE, T2-WI, T2-FLAIR, ADC	Morphological features, Intensity features, Texture features, Histogram features	Random forest	0.96 (AUC)
Park et al. (2020) [9]	Prognostication subtypes model of GBM	T1-CE, T2-FLAIR, DWI, dynamic susceptibility contrast (DSC).	Morphological features, Intensity features, Texture features	Cox regression and LASSO	0.74 (C-index)
Chaddad et al. (2019) [10]	Predicts Survival of IDH1 Wild-Type Glioblastoma	T1-CE, T2-FLAIR	Morphological features, Intensity features, Texture features	Random forest	0.78 (AUC)
Rathore et al. (2018) [11]	Tumor subtypes of GBM with different clinical and molecular characteristics offering prognostic value	T1-WI, T1-CE, T2-WI, T2-FLAIR, DSC-MRI, DTI	Morphological features, Intensity features, Texture features, Histogram features	K-means clustering	0.75 (C-index)
PET/MRI					
Haubold et al. (2020) [12]	Tumor decoding and phenotyping: prediction of 1p/19q co-deletion	T1-CE, ADC, 3D-FLAIR (SPACE)/^18^F-FET	Morphological features, Intensity features, Metabolic features	(1) 1p/19q co-deletion: Random forest (2) MGMT promoter methylation status: SVM	(1) 0.98 (AUC) (2) 0.76 (C-index)
Wang et al. (2020) [13]	Differentiation of radiation necrosis from tumor recurrence	T1-CE, FLAIR/^18^F-FDG & ^11^C-MET PET	Morphological features, Texture features, Metabolic features	LASSO binary logistic regression	0.99/0.91 (AUC)
Lohmann et al. (2018) [14]	Radiomics differentiates radiation injury from recurrent brain metastasis	T1-CE, T2-WI, T2-FLAIR/ ^18^F-FET	Morphological features, Texture features, Histogram features, Metabolic features	Logistic regression	0.96 (AUC)

## Data Availability

The data are in a publicly available dataset that contains no linkage to patient identifiers. https://wiki.cancerimagingarchive.net/display/Public/TCGA-GBM (accessed on 29 March 2021).
